# Artificial intelligence: from the retina to the brain

**DOI:** 10.5935/0004-2749.20210061

**Published:** 2025-02-02

**Authors:** Daniel Araújo Ferraz, Fernanda Tovar-Moll, Rubens Belfort Jr

**Affiliations:** 1 Instituto da Visão, Universidade Federal de São Paulo, São Paulo, SP, Brazil; 2 NIHR Biomedical Research Centre for Ophthalmology, Moorfields Eye Hospital NHS Foundation Trust and University College London Institute of Ophthalmology, London, United Kingdom; 3 Instituto D’Or de Pesquisa e Ensino, Rio de Janeiro, RJ, Brazil

Approximately 50 million people have some form of dementia worldwide, with an incidence of 10 million per year. Global figures are projected to increase to 150 million patients in 2050. Traditionally, patients with suspected dementia are assessed using clinical history, physical examination, questionnaires, and appropriate brain imaging. While the demonstration of misfolded protein through cerebral biopsy is often considered the gold standard for diagnosis, it is rarely executed given its inherent risks^(1)^.

A trend has emerged to adopt less invasive diagnostic tools such as amyloid protein and analysis of other biomarkers in cerebrospinal fluid and imaging techniques such as magnetic resonance imaging (MRI). However, significant logistical barriers remain for conducting these investigations on a large scale. For example, an MRI protocol for neurodegenerative diseases (ND) has a high cost. It can take more than an hour to complete, is restricted to specialist centers, and requires acquisition and interpretation expertise^(2)^. This is in contrast to retinal imaging, which can be procured within seconds.

As part of the central nervous system (CNS), the retina may present an invaluable opportunity to study the pathophysiological changes in ND, such as dementia. Exploiting the homology between the eye and the brain, from their immune privilege status to the microvascular organization, allows leveraging the retinal structure to reflect CNS disease. Translating this rationale into scientific findings has been bolstered by noninvasive imaging techniques such as fundus photography and optical coherence tomography (OCT), facilitating efficient analysis of the retina vascular and neuronal structures. Much of the current work combining ND and retinal morphology is centered around OCT findings such as changes in the retinal nerve fiber and ganglion cell-inner plexiform layers. However, similar associations have also been reported in retinal fundus photography, where changes in vessel caliber and branching indexes have been observed in people with cognitive impairment^(3)^.

Artificial Intelligence (AI) can be considered a beacon of hope to mitigate human-operated health-care weaknesses currently entrenched. We hope this will include an early diagnosis of ND. Within image-driven specialties such as ophthalmology, AI uses convolutional neural networks to harness information from labelled data using pattern recognition. Evidence has been published of its screening capabilities such as diabetic retinopathy, glaucoma, and age-related macular degeneration^(4)^. The value of a potential AI centered approach as a screening tool for ND could be revolutionary; facilitating intervention early in the disease process would lead to improvements in quality-adjusted life-years and reduce mortality and the economic onus of caring for functionally impaired patients. Further work involves integrating these systems into real-world environments, with other prospective non-interventional studies, to answer critical questions regarding automated diagnosis efficacy and, ultimately, their clinical effectiveness.

AI tools are among the most promising solutions to solve modern-day health-care challenges such as aging populations. They could not only enhance the general health of the global community but also alleviate the financial burden of health care. AI is likely to rapidly change the health sphere in the coming decades, although it still poses several challenges that still need to be addressed.
